# Eco-Conversion of Two Winery Lignocellulosic Wastes into Fillers for Biocomposites: Vine Shoots and Wine Pomaces

**DOI:** 10.3390/polym12071530

**Published:** 2020-07-10

**Authors:** Grégoire David, Micaela Vannini, Laura Sisti, Paola Marchese, Annamaria Celli, Nathalie Gontard, Hélène Angellier-Coussy

**Affiliations:** 1 JRU IATE 1208–Univ Montpellier, INRAE, 2 Place Pierre Viala, Bat 31, F-34060 Montpellier 01, France; gregoire.david@supagro.fr (G.D.); nathalie.gontard@inrae.fr (N.G.); 2 Department of Civil, Chemical, Environmental, and Materials Engineering, University of Bologna, Via Terracini 28, 40131 Bologna, Italy; laura.sisti@unibo.it (L.S.); paola.marchese@unibo.it (P.M.); annamaria.celli@unibo.it (A.C.)

**Keywords:** biocomposite, vine shoot, wine pomace, extraction process, mechanical properties

## Abstract

Two winery residues, namely vine shoots (ViSh) and wine pomace (WiPo), were up-cycled as fillers in PHBV-based biocomposites. Answering a biorefinery approach, the impact of a preliminary polyphenols extraction step using an acetone/water mixture on the reinforcing effect of fillers was assessed. Biocomposites (filler content up to 20 wt%) were prepared by melt-mixing and compared in terms of final performance (thermal, mechanical and barrier). It was shown that the reinforcing effect was slightly better in the case of vine shoots, while it was not significantly affected by the pre-treatment, demonstrating that these two winery residues could be perfectly used as fillers in composite materials even after an extraction process to maximize their potential of valorization.

## 1. Introduction 

Agriculture is a huge generator of residues, including a large proportion of solid lignocellulosic biomass, which can be considered as the most highly available renewable resource on Earth at the lowest cost [1]. These residues do not often find a valuable exploitation and they are still sometimes simply burned without valorization [2]. In parallel, the world plastic production is increasing every year, reaching 350 million tons in 2018 [3] and it induces environmental concerns, notably due to its accumulation in nature. These two issues can find a common solution in the development of a generation of biocomposites where a biopolymer matrix is filled with vegetal-derived fillers [4].

In this context, the European project H2020 NoAW [5] aiming to increase the opportunities for agro-waste valorization by enlarging the spectrum of conversion process and product port-folio, is working on the development of eco-efficient products including biocomposites. Specifically, within the different agricultural activities, viticulture has been seen as one of the most spread crops in the world [6] producing a large amount of lignocellulosic residues, such as wine pomaces and vine shoots [7]. Vine shoots (ViSh) are agricultural residues from vine pruning, with approximately 2 tons·ha^−1^ (dry basis) generated per year, corresponding to an annual world production of 15 million tons [7]. They are usually ground and left in the field as organic amendment or burned to prevent the proliferation of phytopathogens [8]. This is due to their very low economic value. The composition of ViSh is characterized by holocellulose (68%), lignin (20%), proteins (5%) and the rest includes small amounts of lipids, polyphenols and ashes [9,10]. Some studies explored the use of ViSh as a source of high added value products, such as polyphenols for their antioxidant properties [11] and especially resveratrol or viniferin [12]. Rayne et al. [13] estimated that the extraction of resveratrol and viniferin from ViSh waste may reach a global economic value of over $30 billion.

Wine pomace (WiPo) is one of the most abundant solid by-products generated during the wine-making process [7]. It corresponds to the solid residue from the pressing of fresh grapes, whether or not fermented [14] and is constituted of stems, skin and seeds. At least 20% of the grapes’ weight becomes pomace, generating more than 10 million tons every year in the world [15]. A small portion of grape pomace follows the traditional ways of valorization, i.e., distillation to produce different types of spirits and liquors, or used as fertilizer after industrial or home composting, or as animal feed [16]. However, the main part of pomaces is traditionally poorly exploited, due to the decontaminating steps that are currently required from environmental policies to remove heavy metals and phytotoxic compounds [17]. Wine pomaces are known to be rich in phenolic compounds, in particular flavonoids, phenolic acids and stilbenoids [18,19], many of which have shown biological activities and have resulted beneficial to human health [20,21]. They therefore are an important exploitable feedstock for the extraction of biomolecules for food, feed, cosmetic and pharmaceutical applications [19].

Very few papers deal with the use of residues stemming from the wine-making process in the preparation of composites. The studies are mainly considering fossil and non-biodegradable polymers as matrices, i.e., polyolefins [22,23,24,25], epoxy resins [26], polystyrene ([27]) or poly(vinyl alcohol) [28], and to a lesser extent, biosourced and biodegradable matrices such as soy flour [28], and very recently poly(lactic acid) [29], poly(butylene succinate) [30] or poly(3-hydroxybutyrate-*co*-3-hydroxyvalerate) (PHBV) [31]. In particular, PHBV is a bacterial polyester presenting the advantage of being potentially prepared from agro-residues. Its mechanical properties are close to those of common fossil-based thermoplastic such as polypropylene (PP). However, as compared to conventional plastics, PHBV is still available at a high price (around 5 €/kg), which limits its use to niche market applications. A larger use of PHBV would require increased production capacities and a decreased production cost and/or finding strategies to decrease the overall cost of the developed materials. The incorporation of low-cost lignocellulosic fillers stemming from agro-residues constitutes one answer to this latter issue [32,33,34], while reducing the environmental impact of the materials and modulating their functional properties. In a previous study, it was shown that PHBV-based composites with ViSh fillers were fully biodegradable in natural conditions [35].

Globally, the introduction of lignocellulosic fillers produced from agro-residues may result in a decrease in ultimate mechanical properties [22,30,31]. This reduction was attributed to a low aspect ratio of particles, e.g., 1.8 in the case of the ViSh fragments obtained by grinding in a granulator equipped with a 2.5 mm sieve [22], or to a poor filler/matrix interface [22,25,30]. A treatment of lignocellulosic fillers, e.g., an alkaline treatment of grape stalks allowing to increase filler surface roughness and crystallinity [27], or the addition of compatibilizers, e.g., the use of maleic anhydride in the case of a system based on high-density polyethylene (HDPE) [22,30], could be useful to enlarge the overall performance of composites. As regards barrier properties, it was shown that the introduction of ViSh particles in PHBV allowed increasing water vapor permeability, which could be interesting for some applications, e.g., horticulture or the packaging of respiring products [31]. A positive effect could also be noticed regarding the thermal stability, with a delay in the thermal degradation, e.g., in the case of ViSh/HDPE [23] or WiPo/PBS [30] systems. It is worth noting that the degradation of mechanical properties was relatively moderate (e.g., 25% decrease for stress at break with 30% of filler content [31]), allowing to conclude on the interest of using such fillers to decrease the overall cost of materials while maintaining or modulating some functional properties.

The implementation of a biorefinery concept by developing cascading processes would be of great interest to give the highest possible value to winery lignocellulosic residues. As mentioned above, the extraction of bioactive compounds is one of the most investigated valorization options for these two agro-residues since the extracted components can have a high market value. At the end, the process still leaves a solid residue, called either exhausted vine shoots or exhausted wine pomaces, that needs to be disposed of. In a cascading logic, an interesting strategy would be to recover the exhausted solid residues and to use them as fillers in biocomposites. Moreover, Krouit et al. [36] showed that the solvent extraction of dry refined softwood fibers appeared as efficient as chemical grafting for compatibilization treatment, meaning to think that the interest of polyphenol extraction before using the biomass for composite applications could be double.

The objective of this work was to assess the possibility of implementing a biorefinery concept by developing cascading processes to give the highest possible value to two winery lignocellulosic residues, i.e., vine shoots and wine pomaces. For this purpose, the reinforcing effect of fillers produced from either vine shoots or wine pomace was compared, by using the fillers prepared either directly from the raw biomass or after a preliminary polyphenol extraction step. The fillers were characterized in terms of biochemical composition, density, morphology, thermal stability and color before being mixed with the PHBV matrix. The study focused on investigating the relationships between the main intrinsic characteristics of the constituents, the structure of the resulting composites and their functional properties (tensile properties, water vapor permeability, thermal stability).

## 2. Materials and Method

### 2.1. Materials

A commercial grade of poly(3-hydroxybutyrate-co-3-hydroxyvalerate) (PHBV) under the reference PHI 002 was purchased from NaturePlast company (Ifs, France). In the form of pellets, this grade contained 1–3 mol% of valerate and 1 wt% of boron nitride as a nucleating agent. It had a true density of 1.24 g∙cm^−3^.

Vine shoots (ViSh) of the Syrah variety (*Vitis vinifera*) L. were kindly provided by Jean-Michel Salmon (UEPR, INRA). They were pruned and collected in 2016 in Gruissan (Languedoc-Roussillon region, France). The fresh vine shoots were air dried during 2 months and then dried in an oven at 60 °C for 24 h before milling. Wine pomace (WiPo) of the Merlot variety (*Vitis vinifera* L.) was provided by InnovEn srl (Verona, Italy) and contained berry skins, seeds, petioles and stalks. The grape was harvested in the year 2016 and the pomace was collected after pressing and wine fermentation. It was frozen and stored at −20 °C the same day as the wine production and then dried in an oven at 60 °C for 24 h before milling.

Acetone from (99.8% of purity) and ethanol (99.9% of purity) were respectively purchased from Biosolve Chimie (Dieuze, France) and Meridis (Montpellier, France). Sodium hydroxide sulfuric acid at 72% was purchased from Sigma Alrich (Darmstadt, Germany). All the chemicals were used without any further purification.

### 2.2. Methods

#### 2.2.1. Extraction of Polyphenols

Before extraction, the ViSh and WiPo were previously milled to obtain particle sizes ranging between 0.5 and 1 mm. These pre-milling conditions were different according to the biomass and adapted according the characteristics of the two residues. The dry pomaces were simply ground in a kitchen blender, while the vine shoots were milled using a cutting mill type SM 300 (Retsch, Haan, Germany) with a 4.0 mm sieve and then 2.0 mm sieve. Solvent-based extractions were carried out with the aim to recover the highest yield of phenolic compounds. The ViSh and WiPo were extracted following the same protocol optimized in a previous study for grape pomaces [37]. Batches of 10 g of substrates were introduced in Pyrex glass reagent bottles with 50 mL of 75% v/v acetone that were incubated for 2 hours at 50 °C in a shaking bath. The bottles were closed to avoid solvent evaporation. After incubation, the liquid extract was separated from the solid residue by centrifugation (5 min, 5000 rpm). The solid residues were dried under the hood overnight, and then dried at 60 °C during 24 h. The liquid extracts were filtered with a 0.45 µm MF—Millipore filter (Merck) stored at −20 °C until further analyses. At the end, 60 g of substrate were extracted, with 6 batches of 10 g. The ViSh and WiPo after extraction were named ViSh-E and WiPo-E, whereas the virgin ViSh and WiPo (without extraction) were named ViSh-V and WiPo-V.

Such a protocol allowed to recover about 20 and 47 mg of polyphenols per gram of the dried ViSh and WiPo, respectively, which was determined by the Folin–Ciocalteu colorimetric method [38]. Spectrophotometrical tests revealed that the extracted polyphenols mainly consisted of flavonoids, flavanols and hydroxycinnamic acids, molecules characterized by high antioxidant capacity [38]. After extraction, around 10% of weight loss (dry matter) was noticed for the fillers.

#### 2.2.2. Production of Fillers

After extraction, the exhausted pomaces and shoots (dried solid residues) were milled with a centrifugal mill ZM 200 (Retsch, Haan, Germany) at 14,000 rpm with 0.5 mm sieve. The samples were stored in a hermetic drum at 23 °C in the presence of silica gel (around 0% of relative humidity, RH) before further analysis. The residues were named ViSh-V and ViSh-E, to refer to vine shoots, virgin and exhausted, respectively; the same setting was used for the wine pomaces with WiPo tag.

#### 2.2.3. Production of Biocomposites

Compounding: before the compounding, the fillers (both virgin and exhausted fillers) and the PHBV pellets were dried under vacuum at 60 °C overnight. The composites were prepared in a Brabender mixer at 180 °C for 6 min at 50 rpm, the mixing time after the filler addition being 3 min. For each of the four types of fillers, three filler contents were considered, i.e., 5, 10 and 20 wt%. The samples were named 5ViSh-V, 10ViSh-V, 20ViSh-V for the composite with virgin ViSh and 5ViSh-E, 10ViSh-E, 20ViSh-E for composite with exhausted ViSh (after extraction), respectively. The same names were used for the wine pomaces with WiPo. The resulting composites were cooled in liquid nitrogen and quickly ground with an Ika M20 Mill (Staufen, Germany). The compounds were dried at 60 °C overnight before using them for a shaping process.

Preparation of films: 3.20 g of the ground compounds were scattered on a Teflon foil (12 × 12 cm) within an aluminum frame (10 × 10 cm), 300 µm thick. A second Teflon foil was arranged on the first one and the resulting foils couple was placed between the plates of a Carver press, then heated at 190 °C under 4.5 bars for 30 s. Then, the films were quickly cooled to room temperature, led to room pressure and finally separated from the Teflon foils.

Preparation of the dog-bone samples. The compounds were injection-molded using a Minijetpro Haake machine (Thermo-Fischer) for tensile tests (dog-bone shape ISO527-2-1BA). The cylinder and mold temperatures were 185 °C and 70 °C, respectively. The injection and holding pressures were 400 and 100 bar, respectively. The injection and holding times were 20 s and 10 s, respectively.

Control materials were produced for each series of biocomposites. PHBV ref1 was the control for the ViSh-based composites, while PHBV ref2 was the control for the WiPo-based composites.

#### 2.2.4. Scanning Electron Microscopy (SEM)

SEM observations were done with a Hitachi S4800 scanning electron microscope (Technology platform of IEM Laboratory of the Balard Chemistry pole, Montpellier, France) with an acceleration voltage of 2 kV after a coating with Pt by cathode pulverization. The observations were realized with the help of Didier Cot (IEM, Montpellier). For the particle observation, a solution of 1.0 g·L^−1^ of particles in pure ethanol was first prepared, then 50 µL of this solution was dropped on brass support. This solution was preferred to a direct spray of the particles. In case of composite films, the cross-section was observed after the tensile tests or after the cryo-fracture in the liquid nitrogen.

#### 2.2.5. Laser Granulometry

Filler size distribution (apparent diameter of particles), median apparent diameter (d50) and the span values were determined using a laser granulometer in the wet mode (Malvern Mastersizer 2000 Instrument Ltd., United Kingdom), by dispersing the particles in ethanol 95% (v/v). The span values were calculated as the difference between the 90th and the 10th percentile, divided by the median apparent diameter ((d90-d10)/d50). Measurements were done in triplicate.

#### 2.2.6. Color

The color attributes of each ViSh- and WiPo-based fractions and biocomposites were measured with a colorimeter CR-410 (Minolta), using the CIELAB color system (L*, a*, b*). L* represents the luminance, ranging from 0 (black) to 100 (white), while the two chromatic components a* (from green to red) and b* (from blue to yellow), range from −120 to +120. The total color difference (ΔE) was calculated following the standard ASTM D2244 [39] (Equation (1)):

ΔE = [(L* − L_0_*)^2^ + (a* − a_0_*)^2^ + (b* − b_0_*)^2^]^0.5^ (1)

where L*, a*and b* represent the color components of each sample. The references were respectively 99.5% pure cellulose BE 600–10TG (Arbocel, France) for the fillers, and the neat polymer matrix for the biocomposites. All the measurements were carried out with 5 repetitions.

#### 2.2.7. Biochemical Composition

The ash content was determined in triplicate from the residue after thermogravimetric analysis (Mettler TGA2, Schwerzenbach, Switzerland) at 800 °C under air. Klason lignin was determined by mass deduction after double hydrolysis in acidic conditions. Moreover, 80 mg of dry extracted biomass was put in 0.85 mL of sulfuric acid 72% and stirred manually for 1 h at room temperature. Then, 23.8 mL of ultrapure water was then added, and the mixture was heated and stirred at 121 °C during 1 h in a sealed flask. Subsequently, the hydrolysates were filtered (10 μm), washed by water, and dried at 105 °C overnight. The ash also contained in the residues were subtracted to know the lignin content. The analyses were done in duplicate.

#### 2.2.8. Differential Scanning Calorimetry

Calorimetric analysis was carried out by means of a Perkin Elmer DSC6 calorimeter, calibrated with high-purity standards. The measurements were performed under a nitrogen flow. The thermal treatments used were as follows: first scan, from 30 to 210 °C at 20 °C·min^−1^ and 1 min of isotherm at 210 °C; cooling scan, from 210 to 0 °C at 20 °C·min^−1^ and 1 min of isotherm; second scan, from 0 to 210 °C at 20 °C·min^−1^. The percentage of crystallinity (Xc) was calculated by using the following Equation (2):

(2) $$\mathrm{Xc}=\frac{\Delta \mathrm{Hm}}{\Delta H{}^{\circ}m\times \left(1-w\right)}\times 100$$

where ΔH°m = 146.6 J·g^−1^ is the enthalpy corresponding to the melting of a 100% crystalline PHB sample while the term 1-w represents the biopolymer weight fraction in the composite [40].

#### 2.2.9. Thermogravimetric Analysis

Thermogravimetric analysis (TGA) under nitrogen flow (40 mL∙min^−1^) was carried out by using a Mettler TGA2 apparatus (Schwerzenbach, Switzerland) equipped with a XP5U balance (precision of 0.0001 mg). For each measurement, about 40 mg of material were heated from 40 °C to 600 °C at 10 °C∙min^−1^. The onset degradation temperature (T_onset_) was corresponding to the interception of the tangent drawn at the inflection point of the decomposition step with the horizontal zero-line of the TGA curve. The temperature of degradation (T_deg_) corresponded to the temperature at which the degradation rate was maximum.

#### 2.2.10. Mechanical Properties

Tensile tests were conducted using an INSTRON 5966 (Instron, Turin, Italy) series test apparatus equipped with a 10 kN load cell. The cross-head speed was set at 5 mm∙min^−1^, and the test was performed at room temperature. The reported data are the average values of at least ten determinations for each sample.

#### 2.2.11. Water Vapor Permeability (WVP)

Water vapor permeability of films (mol∙m^−1^∙s^−1^∙Pa^−1^) was determined at 23 °C using a gravimetric method [41]. The composite films (five repetitions) were hermetically sealed with Teflon seal in glass permeation cells containing distilled water. These cells were placed in a desiccator containing silica gel (RH around 3%). They were weighed using a four-digit balance (BALCO–Type LX 220A, Switzerland) at a regular interval during ten days. Water vapor permeability was calculated from the following equation (Equation (3)):

(3) $$\mathrm{WVP}=\frac{S\;\times \;e}{3600\times \;A\;\times \;\Delta P\;\times {\;M}_{H_{2}O}}$$

where S is the slope of the weight change from the straight line (g·h^−1^), A is the permeation area (m^2^), e is the average specimen thickness (m), ΔP is the water vapor pressure differential (Pa), and M_H2O_ is the molar mass of water (g·mol^−1^).

## 3. Results and discussion

### 3.1. Intrinsic Properties of Wine Pomace and Vine Shoots Particles

#### 3.1.1. Particle Biochemical Composition

Table 1 reports some data concerning the chemical composition of vine shoots and wine pomace. The content of Klason lignin as an acid-insoluble residue was formally determined following a two-step hydrolysis. The Klason lignin content was respectively of 19.4% and 34.6% for the vine shoots (ViSh) and wine pomace (WiPo), which was common for such residues [7,42,43,44]. In the case of WiPo, the extraction treatment resulted in an increase in the Klason lignin content from 34.6% for WiPo-V (virgin) up to 41.7% for WiPo-E (exhausted, after extraction). This could be explained by the fact that during the extraction, some sugars are also extracted, resulting in a proportional increase in lignin content. This was not observed for ViSh-E, displaying a slightly lower value of lignin content than ViSh-V. It was explained by the fact that vine shoots contained much less soluble sugars than pomace, and condensed tannins were possibly extracted whereas they were included in lignin content for ViSh-V. It is worth noting that ash content was higher in pomace than in vine shoots.

Such differences in the chemical composition between the shoots and pomace may result in differences in the intrinsic physico-chemical properties of biomasses, including color, mechanical properties, capability to be milled, and thermal stability.

#### 3.1.2. Particle Color

The ViSh particles were light brown whereas the WiPo particles were dark red. They were both derived from red wine varieties, however, the red color was not visible in the vine shoots. The color was quantitatively assessed by the (L*, a*, b*) values (Table 1). The gap ΔE with pure cellulose was clearly more important for WiPo (ΔE = 41.0) than for ViSh (ΔE = 23.2). The darker color of wine pomaces was due to the higher content in polyphenols and lignin [45]. The extraction step did not result in a similar and clear change in color for ViSh and WiPo (Table 1). As a whole, the particle color does not prevent the exploitation of these residues as fillers in polymeric matrixes, mainly if the final applications do not require a high aesthetic quality.

#### 3.1.3. Particle Morphology, Size and Density

**Figure 1:**
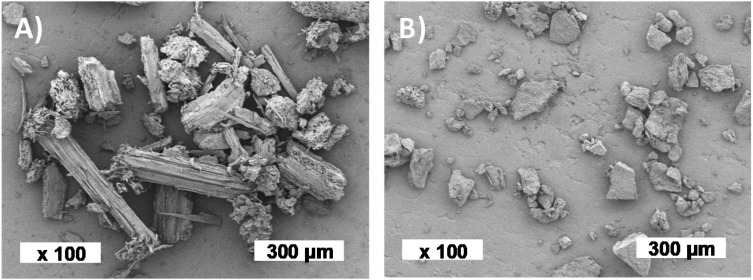
SEM images of (**A**) the vine shoots and (**B**) the wine pomace fillers.

The milled particles were poorly elongated with a low aspect ratio visually deduced from Figure 1. ViSh particles seem more elongated than WiPo with a more rectangular shape and a more fibrous structure (Figure 1A). Numerous small and more spherical particles were also observed. On the contrary, WiPo were close to square-like particles with an aspect ratio close to 1 (Figure 1B). Despite the fact that the same grinding process was used, ViSh and WiPo particles did not display exactly the same size (Table 2), with a volume median equivalent diameter d50 of 143 µm and 114 µm for ViSh-V and WiPo-V, respectively. This highlighted that WiPo was more fragile, or in other words less resistant to fractionation forces. ViSh-V (1.449 g∙cm^−3^) are a little denser than WiPo-V (1.420 g∙cm^−3^) due to different microstructures [7]. After extraction, the density did not change or only by very slightly in both cases, due to the revelation of microporosities (1.445 and 1414 g∙cm^−3^ for ViSh-E and WiPo-E, respectively).

**Figure 2:**
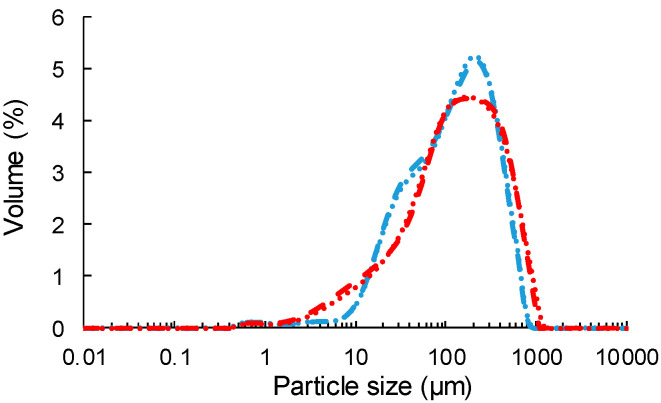
Particle size distribution for ViSh-V (- -), ViSh-E (**∙ ∙**), WiPo-V (- -) and WiPo-E (**∙ ∙**) in volume.

As already shown by the SEM observations, the particle size distributions were characterized by a high size polydispersity, even higher for the ViSh samples, with span values of 3.7 for ViSh and 3.0 for the WiPo samples. This was ascribed to the complex heterogeneous structure of the lignocellulosic biomass. Several populations can thus be seen within each sample size distribution (Figure 2). The WiPo particles seem to be constituted of two fractions, with a respective median size of 200 µm and 50 µm. ViSh, on the other hand, slightly displays two fractions: a major one, centered on 180 µm, and a minor one, around 20 µm. The repartition of these different fractions could be ascribed to differences in tissue grindability within the complex lignocellulosic structures that are the studied fillers [46]. It was checked that the extraction process had no significant impact on the morphology nor on the particle size (Table 2). Considering these characteristics, both the virgin and exhausted residues turned out to be successfully usable as fillers in a polymeric matrix, however, the shoots being preferable in reason of their elongated shape, which could interfere with mass transfer [47] and mechanical properties [22].

#### 3.1.4. Thermal Stability of the Fillers

**Figure 3:**
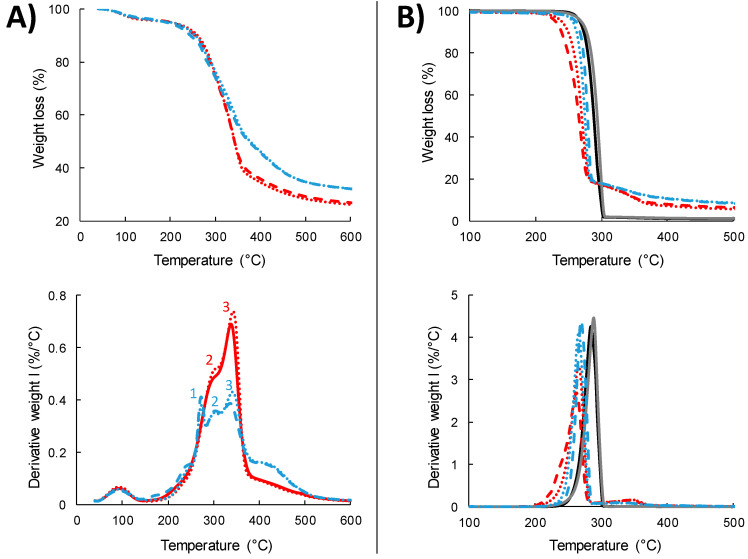
Thermogravimetric analysis of (**A**) the fillers: ViSh-V (- -), ViSh-E (**∙ ∙**), WiPo-V (- -) and WiPo-E (**∙ ∙**) and (**B**) the composites: PHBV-ref 1 (**―**), PHBV-ref2 (**―**) 20ViSh-V (- -), 20ViSh-E (**∙ ∙**), 20WiPo-V (- -) and 20WiPo-E (**∙ ∙**) under nitrogen.

The thermal stability of the fillers is of great importance for biocomposite manufacturing, since it affects their processability and their overall thermal resistance. For this reason, the thermal stability of the produced ViSh and WiPo particles was evaluated under inert conditions by thermogravimetric analysis (TGA) in the temperature range from 20 °C to 600 °C. The thermograms of weight loss and derivative weight loss of both virgin and exhausted particles are displayed in Figure 3A. In addition, the temperatures of the degradation peaks, indicated in Figure 3A with the numbers 1–3, are summarized in Table 3. The two biomasses, i.e., ViSh and WiPo, exhibited a similar thermal degradation pattern, which was characteristic of lignocellulose [30,48]. The first weight loss between 40 and 130 °C corresponded to the evaporation of water. The water amount was similar for all the samples, around 3.8% (wet basis). The second step started around 170 °C and extended to 500 °C. It corresponded to the degradation of organic compounds, including extracted molecules, hemicellulose, cellulose and lignin.

In the case of ViSh, the peaks associated to the degradation of hemicellulose and cellulose were quite well distinguished, with a temperature of maximum rate of degradation of hemicellulose (T_deg_2) at 302 °C and a temperature of maximum rate of degradation of cellulose (T_deg_3) at 338 °C, respectively (Table 3). The last shoulder corresponded to the degradation of lignin that is known to occur over a large range of temperature. From 500 °C, a passive pyrolysis step was characterized by a low and a continuous mass loss rate corresponding to the end of lignin degradation as well as char formation and rearrangement. The residue at 600 °C represented 26.5% of the ViSh. This profile was similar to a stone-rich fraction of olive pomace [49].

In the case of WiPo, the TGA pattern displayed numerous degradation steps, showing that the biomass was more complex in terms of composition. As compared to ViSh, the intensities of the peaks associated to the degradation of hemicellulose and cellulose were decreased by about 22% and 40%, respectively, while the intensity of the peak associated to the degradation of lignin was almost doubled (Figure 3A), which was in accordance with the results of chemical composition. In the WiPo analysis, a first peak was visible, with a maximum rate of degradation at 273 °C (T_deg_1) and a shoulder was around 240 °C. They are probably associated to the degradation of pectin or non-structural sugars.

The polyphenol extraction did not change the thermal degradation profiles of ViSh or WiPo. However, small differences could be observed regarding the intensities of the peaks on the derivative curves. The temperatures of the maximum degradation rate of cellulose (T_deg_3) slightly shifted to higher temperatures, i.e., from 337.9 to 342.4 °C for ViSh and from 336.3 to 341.3 °C for WiPo fillers, respectively. The increase in the peak intensity shows that they are more cellulose in proportion in the two fillers. The extraction moderately improved the material stability, with onset temperatures at 7 and 8 °C higher than the virgin fillers for ViSh-E and WiPo-E, respectively. The same results were observed for the enzymatically-treated rice endosperm [50].

Therefore, the thermal data confirmed that the investigated fillers were stable until 200 °C and all their processing must be performed under this temperature. Moreover, the matrix choice was guided by this information, because the melt compounding requires a polymer with a melting temperature lower than 200 °C.

### 3.2. Impact of Biomass Origin and Treatments on the Properties of Biocomposites

#### 3.2.1. Visual Appearance of Films

The color of the materials could be an important parameter for consumer acceptability. PHBV films were translucent and were characterized by a light beige color. The introduction of ViSh or WiPo particles resulted in darker and more brown materials, highlighted by a significant decrease in L*, an increase in a* and a decrease in b* (Table 4). This evolution was even more pronounced in the case of WiPo particles, with even more brown and darker composite materials. As expected, the resulting color of biocomposites was dependent on the color of fillers. However, it is worth noting that the differences in color (ΔE values) observed for the ViSh and WiPo fillers were less marked when incorporated in the PHBV matrix (Table 4).

As expected, increasing the filler content also resulted in a continuous decrease in L*, an increase in a* and a decrease in b*. As regards the effect of extraction, the films were a little bit lighter (increase in L*, related to higher values of L* in the case of exhausted samples). Food trays or horticulture pots are possible applications that do not necessarily need transparent or light-colored materials.

#### 3.2.2. Observation of the Microstructure by SEM

**Figure 4:**
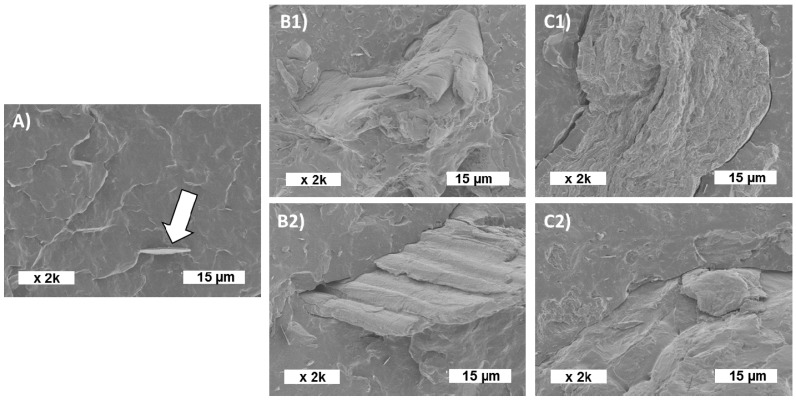
SEM observation of the cross section of composites: (**A**) neat PHBV (white arrow shows boron nitride), (**B1**) 20ViSh-V, (**B2**) 20ViSh-E, (**C1**) 20WiPo-V and (**C2**) 20WiPo-E.

The cryo-fractured surfaces of composite films were observed by SEM (Figure 4). Neat PHBV (Figure 4A) displayed a quite smooth surface with the presence of boron nitride (white arrow) used as a nucleating agent in the PHBV grade PHI002. The incorporation of fillers led to a rough section, with the evidence of well embedded fillers (Figure 4B,C). No large interfacial holes or voids were visible, whatever the filler type and treatment, which allowed assuming that the filler/matrix interfacial adhesion was good.

#### 3.2.3. Differential Scanning Calorimetry (DSC) characterization

The melting and crystallization temperatures and enthalpies, as well as crystallinity degrees, were determined from the DSC thermograms and reported in Table 5. PHBV is a semi-crystalline polymer characterized by a melting peak at about 172 °C and a crystallization peak, visible during the cooling scan, at 113 °C. The crystallization and melting enthalpies are high (ΔHc = 72 J·g^−1^; ΔHm = 83 J·g^−1^ about), corresponding to a crystallinity of about 57%, calculated according to Equation (2). Moreover, the polymer was able to fully crystallize during the cooling step, due to the presence of inorganic nucleating agents (boron nitride) in the formulation.

The addition of the lignocellulosic residues into the PHBV matrix entailed a slight decrement in the crystallization temperatures during the cooling scan. In particular, Tc went from 113 °C for PHBV to 108 °C for the sample containing 20 wt% of WiPo (either virgin or exhausted). This behavior indicated that the wine pomaces acted as physical hindrances to the chain mobility, slightly slowing down the crystallization process from the melt. In the case of ViSh, such delay in crystallization was not observed. In any cases, the polymeric matrix completely crystallized during the cooling step from the melt and the subsequent melting process was very similar to that of the sample without fillers. As expected, the melting and crystallization enthalpies, related to the melting and crystallization of PHBV crystals, decreased in the composites, proportionally to the filler content.

According to Equation (2), the presence of fillers did not significantly impact the PHBV crystallinity (X_c_), always ranging from 55 to 58%. In the case of composites with exhausted pomaces, the X_c_ mildly increased, indicating a slight nucleating effect imparted by these fibers [40].

On the whole, the DSC data show that the PHBV thermal characteristics are basically maintained, indicating that the biocomposites can be subjected to the same processing of the PHBV matrix.

#### 3.2.4. Thermal Stability

The thermal stability of materials is an important criterion since they can be submitted to heating cycles during their service use or end-of-life treatment. The thermogravimetric analysis data carried out on the samples are reported in Table 5 and Figure 3B shows the TGA curves for the composites filled with 20 wt% of particles. The PHBV matrix degraded in a single step, with a thermal degradation beginning around 280 °C and a maximum rate of degradation between 291 and 299 °C depending on the batch. The accepted mechanism for the PHBV degradation consists in a random breakage of ester bonds to vinyl ester and carboxyl groups through a single step [51,52]. The PHBV ref1 and ref 2 displayed some differences concerning the temperatures of degradation with 8 °C of deviation for T_onset_ and T_deg_, demonstrating the necessity of systematically using a reference at each production.

The biocomposites presented a lower initial degradation temperature with respect to PHBV and a second degradation stage, due to the fillers’ decomposition. The extent of this additional degradation was proportional to the filler content. In any case, the addition of vine shoots or wine pomaces decreased the thermal stability of the materials, which could also be ascribed to a pro-degrading effect of fillers, promoting the PHBV chain scission. Composites with ViSh degraded at a temperature lower than the composites with WiPo. ViSh seems to favor the polymer degradation. A different slope for 20ViSh-V was noticed compared to 20WiPo-V.

Moreover, the extraction step had a different effect on the composite thermal stability depending on the nature of the residue. In the case of ViSh, the thermal stability of ViSh-E-based composites was improved compared to the ViSh-V-based composites. This is due to the higher thermal stability of ViSh-E particles. In the case of WiPo, the WiPo-E-based composites degraded at lower temperatures than the WiPo-V-based composites, in spite of the higher thermal stability of the WiPo-E fillers. WiPo contained a higher percentage of polyphenols than ViSh which was able to protect the matrix and delay the composites’ degradation, in reason of their antioxidant capability. The extraction of polyphenols causes a decrement of the stability of the composite. As shown in the *Supplementary results*, the molecular weight did not decrease with the addition of fillers and cannot explain the decrease in the degradation temperatures.

#### 3.2.5. Mechanical Properties

The results of the tensile tests on biocomposites with an increasing filler content are presented in Table 6. PHBV exhibited a rigid behavior with a tensile strength of around 40 MPa, a Young’s modulus of 2.2 GPa and an elongation at a break lower than 5% (3.5 and 4.1% depending on the reference).

The addition of fillers led to a reduction of both tensile strength and the strain at break. The similar evolution of mechanical properties was commonly noticed in all the studies dealing with biocomposites [22]. In the present work, this reduction is moderated. The strain at break decreased by 37% for the 20WiPo-V and by 25% for 20ViSh-V. The difference between the formulations was more drastic concerning the stress at break. The stress at break value of 20ViSh-V was only reduced by 12% compared to the control PHBV, whereas with 20WiPo-V, it had a value that was 41% lower. Thus, ViSh seemed to display a better reinforcing effect than WiPo. This could be ascribed to a better affinity with the polymer matrix (but not evidenced on SEM pictures), a more elongated shape of ViSh particles compared to WiPo and/or higher intrinsic mechanical properties of ViSh particles due to a higher content of holocellulose. The rigidity of materials was slightly increased by the addition of ViSh fillers, due to a higher stiffness than the matrix. On the contrary, the addition of pomaces did not change the Young’s modulus of the corresponding materials.

No significant impact of the extraction on the composites materials was observed, meaning that the extraction did not considerably affect the overall mechanical behavior of the composites. It can be concluded that the extraction of high value biomolecules from agro-residue particles could be an interesting strategy since it did not alter the mechanical properties of the resulting composites.

#### 3.2.6. Water Vapor Permeability

**Figure 5:**
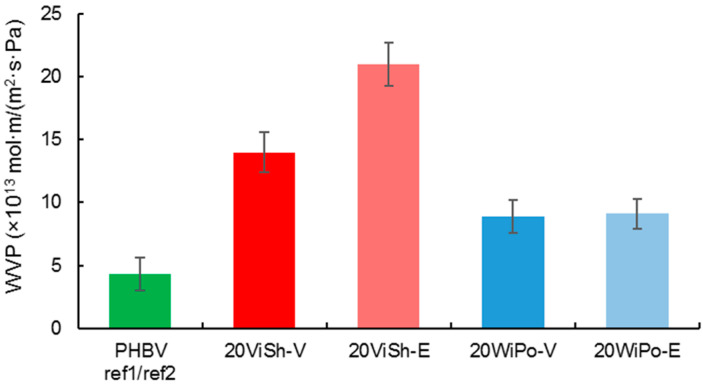
Water vapor permeability of the ViSh and WiPo composites filled with 20 wt% of particles.

The water vapor permeability of the neat PHBV film, 4.3 ± 1.3 ×10^13^ mol·m/(m^2^·s·Pa) was close to the previous results reported in the literature [31,53] (Figure 5). The two controls, i.e., PHBV ref1 and ref2, gave the same results, meaning that the previously observed variability of the mechanical and thermal properties did not impact the water vapor permeability. The introduction of the 20 wt% of ViSh or WiPo fillers led to an increase in the water vapor permeability. The water vapor permeability of 20ViSh-V and 20WiPo-V were 14 ± 1.6 and 8.9 ± 1.3 mol·m/(m^2^·s·Pa), respectively. In the case of composites with 20 wt% of ViSh fillers, the WVP was twice as high compared to the composites previously observed by David et al. [31]. This was due to the higher size of the fillers, i.e., with a d50 of 150 μm instead of 50 µm (aggregation, more defects, edge effects even more favored by a lower thickness of the films [34]).

The increase in WVP was more important with the ViSh fillers, with an increase in more than and times for 20ViSh-V. The rise in WVP is generally explained by the increase in water vapor sorption due to the hydrophilic character of lignocellulosic fillers compared to PHBV [45,54]. The lower increase in WVP in the case of WiPo-based composites may be due to a lower water vapor sorption of the WiPo particles, owing to the higher lignin content. Lignin is indeed known to be highly hydrophobic [55].

The extraction step resulted in a more pronounced increase in WVP in the case of ViSh-based composites, while it did not impact the behavior of WiPo-based composites. This would mean that the extraction process of polyphenols resulted in an increase in the hydrophilicity and thus water vapor sorption of ViSh-E while it did not impact the water sorption behavior of WiPo-E. This is related to the significant increase in holocellulose content in ViSh-E (as highlighted by the TGA measurements) proportionally to the decrease in extractive content, cellulose being the most hydrophilic constituent of lignocellulose [55]. In the case of WiPo-E, the high Klason lignin content was assumed to mask any possible increase in hydrophilicity of WiPo.

Due to their increased WVP with respect to the PHBV matrix, the obtained biocomposites, and particularly those based on vine shoots, could find application in the horticulture sector or in the packaging area of respiring products.

## 4. Conclusions

The present work aimed to evaluate the different exploitation routes of winery residues, according to the principles of the circular economy that wants to valorize all the residues produced along a value chain. In particular, the residues based on vine shoots (ViSh) and wine pomaces (WiPo) were investigated as fillers in polymeric composites. It was shown that both residues were thermally stable under 200 °C and can be thus processed to prepare PHBV composites by melt mixing. The composites were produced with a filler content from 5 to 20 wt%. The SEM observations of the cryo-fracture of the composites displayed the same filler/matrix interfacial adhesion which was quite good. The addition of fillers decreased the thermal stability, especially in the case of ViSh. The composites had lower tensile properties compared to the neat PHBV. This decrease was moderated especially for PHBV/ViSh composites which displayed a higher mechanical performance than the PHBV/WiPo composites. As a result, the ViSh and WiPo particles can be perfectly used as alternative fillers composite materials in an amount up to 20 wt%.

In addition, the virgin and exhausted ViSh and WiPo were used as fillers to study the effect of polyphenol extraction with an acetone/water mixture. From the present results, it can be considered that a first step of polyphenol extraction before using these agro-residues as a filler does not significantly alter the main properties of the composites. Only the WVP of the composites with 20 wt% of ViSh-E was increased by 50%, which may be interesting for food packaging application. Thus, in a biorefinery approach it would be probably worth extracting polyphenols before using these winery residues as a filler for composites to exploit their full potential.

## Figures and Tables

**Table 1:** Klason lignin and ash content and the color attributes of fillers.

	Klason Lignin (%)	Ash (%)	L*	a*	b*	ΔE
**Cellulose**	-	-	76.8 ± 0.2	1.3 ± 0.1	5.1 ± 0.1	-
**ViSh-V**	19.4 ± 0.5	3.9 ± 0.2	62.2 ± 0.5	6.8 ± 0.1	22.2 ± 0.3	23.2 ± 0.6
**ViSh-E**	17.7 ± 0.5	4.7 ± 0.5	65.1 ± 0.4	7.0 ± 0.1	22.8 ± 0.1	21.9 ± 0.2
**WiPo-V**	34.6 ± 0.9	5.5 ± 0.7	37.9 ± 0.2	12.7 ± 0.1	11.7 ± 0.1	41.0 ± 0.2
**WiPo-E**	41.7 ± 1.0	6.6 ± 0.2	35.9 ± 0.1	11.7 ± 0.0	12.0 ± 0.0	42.7 ± 0.1

**Table 2:** Filler density and apparent diameter (in volume).

Sample	Density (g∙cm^−3^)	d10 (µm)	d50 (µm)	d90 (µm)	Span
ViSh-V	1.449 ± 0.002	17 ± 1	143 ± 11	539 ± 67	3.7
ViSh-E	1.445 ± 0.002	20 ± 1	143 ± 9	569 ± 26	3.9
WiPo-V	1.420 ± 0.003	22 ± 1	114 ± 4	363 ± 15	3.0
WiPo-E	1.414 ± 0.002	21 ± 1	121 ± 5	370 ± 16	2.9

**Table 3:** Results from the thermogravimetric analysis under N_2_ of the ViSh and WiPo fillers.

Sample	T_onset_ (°C)	T_deg_1 (°C)	T_deg_2 (°C)	T_deg_3 (°C)	Residues at 600 °C (%)
ViSh-V	206 ± 1	-	302.4 ± 0.4	337.9 ± 0.3	26.5 ± 0.7
ViSh-E	218 ± 1	-	305.6 ± 0.6	342.4 ± 0.3	26.2 ± 0.1
WiPo-V	198 ± 1	272.9 ± 0.3	303.6 ± 0.1	336.3 ± 0.3	32.2 ± 0.1
WiPo-E	215 ± 1	272.7 ± 0.1	302.6 ± 0.3	341.3 ± 0.2	32.2 ± 0.1

**Table 4:** Color attributes of the PHBV-based composite films.

	L*	a*	b*	ΔE
PHBV	82.1 ± 0.6	1.9 ± 0.2	10.4 ± 0.8	-
5ViSh-V	68.8 ± 0.9	7.8 ± 0.2	10.1 ± 0.5	14.5 ± 0.9
10ViSh-V	63.6 ± 0.8	8.6 ± 0.1	7.1 ± 0.6	19.9 ± 0.8
20ViSh-V	59.5 ± 0.3	9.0 ± 0.2	3.8 ± 0.2	24.6 ± 0.3
5ViSh-E	70.9 ± 0.5	7.4 ± 0.2	11.5 ± 0.6	12.5 ± 0.5
10ViSh-E	65.9 ± 0.6	8.6 ± 0.1	10.0 ± 0.6	17.5 ± 0.6
20ViSh-E	61.5 ± 0.5	9.4 ± 0.2	6.0 ± 0.6	22.3 ± 0.5
5WiPo-V	63.4 ± 0.4	8.1 ± 0.1	5.1 ± 0.4	20.4 ± 0.4
10WiPo-V	57.6 ± 0.4	7.8 ± 0.2	1.9 ± 0.4	26.5 ± 0.5
20WiPo-V	55.3 ± 0.2	5.6 ± 0.1	−1.7 ± 0.1	29.6 ± 0.2
5WiPo-E	66.4 ± 0.4	8.4 ± 0.2	6.1 ± 0.2	17.5 ± 0.5
10WiPo-E	60.4 ± 1.6	8.7 ± 0.2	2.8 ± 0.5	23.9 ± 1.6
20WiPo-E	56.1 ± 0.2	7.1 ± 0.0	−1.0 ± 0.0	28.8 ± 0.2

**Table 5:** Thermal properties of the ViSh- and WiPo-based composite films.

Samples	Tm^a^ (°C)	ΔHm^a^ (J/g)	Tc^b^ (°C)	ΔHc^b^ (J/g)	Tm^c^ (°C)	ΔHm^c^ (J/g)	Xc^c^ (%)	T_onset_^d^ (°C)	T_deg_^d^ (°C)
PHBV ref 1	176	82	113	72	172	83	57	276	291
5ViSh-V	174	74	113	68	171	78	56	258	274
10ViSh-V	175	81	112	65	170	75	57	271	281
20ViSh-V	173	60	111	56	170	65	55	248	268
5ViSh-E	173	74	113	68	171	79	57	272	283
10ViSh-E	174	78	112	66	171	76	58	276	284
20ViSh-E	173	65	112	58	170	66	56	256	271
PHBV ref 2	172	83	113	74	171	85	58	284	299
5WiPo-V	175	78	111	69	172	79	57	283	293
10WiPo-V	174	70	110	64	172	74	56	273	282
20WiPo-V	174	64	108	57	170	67	57	272	280
5WiPo-E	174	86	111	71	172	83	60	276	290
10WiPo-E	174	71	110	65	171	75	59	275	287
20WiPo-E	173	71	108	58	170	69	59	265	276

^a^ determined by differential scanning calorimetry (DSC) during the first heating scan; ^b^ determined by DSC during the cooling scan; ^c^ determined by DSC during the second heating scan; ^d^ determined by thermogravimetric analysis (TGA) under N_2_ flux, by heating at 10 °C/min.

**Table 6:** Mechanical properties of the ViSh- and WiPo-based composite-injected molded samples.

Samples	Young’s Modulus (MPa)	Tensile Strength (MPa)	Strain at Break (%)
PHBV ref 1	2160 ± 20	40.4 ± 1.6	3.5 ± 0.3
5ViSh-V	2212 ± 40	40.3 ± 0.9	3.7 ± 0.1
10ViSh-V	2300 ± 42	38.3 ± 0.3	3.0 ± 0.2
20ViSh-V	2500 ± 29	35.5 ± 0.6	2.6 ± 0.1
5ViSh-E	2234 ± 33	39.2 ± 1.1	3.5 ± 0.3
10ViSh-E	2264 ± 58	37.3 ± 0.9	3.1 ± 0.2
20ViSh-E	2433 ± 43	34.9 ± 0.6	2.6 ± 0.2
PHBV ref 2	2182 ± 55	41.6 ± 1.1	4.1 ± 0.2
5WiPo-V	2259 ± 24	37.2 ± 1.1	3.1 ± 0.2
10WiPo-V	2260 ± 24	34.4 ± 0.6	2.9 ± 0.1
20WiPo-V	2162 ± 83	24.7 ± 1.5	2.6 ± 0.2
5WiPo-E	2197 ± 100	37.4 ± 1.2	3.5 ± 0.1
10WiPo-E	2207 ± 66	33.0 ± 1.5	3.1 ± 0.2
20WiPo-E	2187 ± 43	26.2 ± 0.6	2.5 ± 0.2

## Supplementary Materials

The following are available online at https://www.mdpi.com/2073-4360/12/7/1530/s1, Table S1: the molecular weight data measured by gel permeation chromatography (GPC) for the indicated samples and polydispersity (PD).
