# Supplementary material for: Eco-Conversion of Two Winery Lignocellulosic Wastes into Fillers for Biocomposites: Vine Shoots and Wine Pomaces
Source: Polymers (Basel). 2020 Jul 10;12(7):1530. doi: 10.3390/polym12071530 (PMC7408111; doi:10.3390/polym12071530)
Supplement: Supplementary file 1 [file polymers-12-01530-s001.pdf]

polymers

MDPI

Article

Eco-Conversion of Two Winery Lignocellulosic Wastes into Fillers for Biocomposites: Vine Shoots and Wine Pomaces

Grégoire David [1], Micaela Vannini [2,*], Laura Sisti [2], Paola Marchese [2], Annamaria Celli [2], Nathalie Gontard [1] and Hélène Angellier-Coussy [1,*]

1 JRU IATE 1208 – Univ Montpellier, INRA, 2 Place Pierre Viala, Bat 31, F-34060 Montpellier 01, France. gregoire.david@supagro.fr (G.D), nathalie.gontard@inrae.fr (N.G), helene.coussy@umontpellier.fr (H.A.C)

2 Dept. of Civil, Chemical, Environmental, and Materials Engineering, University of Bologna, Via Terracini 28, 40131, Bologna, Italy. micaela.vannini@unibo.it (M.V), laura.sisti@unibo.it (L.S), paola.marchese@unibo.it (P.M), annamaria.celli@unibo.it (A.C)

* Correspondence: helene.coussy@umontpellier.fr (H.A.C), +33 (0)499612432; micaela.vannini@unibo.it (M.V), +39 (0)51 2090359.

Gel permeation chromatography (GPC)

GPC measurements were performed at 30°C on a GPC Hewlett Packard Series 1100 using a PL gel 5 μm Minimixed-C column with chloroform as an eluent with a 0.3 mL/min flow; the Refractive Index detector was used and a calibration plot was constructed with monodisperse polystyrene standards. The composite samples were dissolved in a mixture of $CHCl_3$/1,1,1,3,3,3-hexafluoro-2-propanol (HFIP) 95/5 v/v and filtered on a Teflon syringe filter with a pore size of a 0.45 μm Teflon sieve to eliminate the insoluble residue.

It is notable to observe that the molecular weight of the PHBV matrix did not change with respect to the nature of the filler nor the filler content. Therefore, the changes of the properties of the composites, in particular the thermal stability, with respect to those of the polymeric matrix cannot be explained by modifications in the molecular weight. See Table 1S below:

Table 1. S: the molecular weight data measured by gel permeation chromatography (GPC) for the indicated samples and polydispersity (PD).

	Mn $\cdot 10^{-3}$	Mw $\cdot 10^{-3}$	PD
PHBV ref 1	98.5	216	2.2
5ViSh-V	79.5	212	2.7
10ViSh-V	88.0	210	2.4
20ViSh-V	92.1	219	2.4
5ViSh-E	93.5	209	2.2
10ViSh-E	89.6	217	2.4
20ViSh-E	90.8	207	2.3
PHBV ref 2	83.4	201	2.4
5WiPo-V	98.4	212	2.2
10WiPo-V	92.8	224	2.4
20WiPo-V	88.6	218	2.5
5WiPo-E	94.0	208	2.2
10WiPo-E	88.4	206	2.3
20WiPo-E	92.6	224	2.4

© 2020 by the authors. Submitted for possible open access publication under the terms and conditions of the Creative Commons Attribution (CC BY) license (http://creativecommons.org/licenses/by/4.0/).
